# COVID-19 Crisis Creates Opportunity towards Global Monitoring & Surveillance

**DOI:** 10.3390/pathogens10030256

**Published:** 2021-02-24

**Authors:** Ahmed Donia, Sammer-ul Hassan, Xunli Zhang, Lamiaa Al-Madboly, Habib Bokhari

**Affiliations:** 1 Biosciences Department, Faculty of Science, Comsats University Islamabad, Islamabad 45550, Pakistan; ahmeddonia123@yahoo.com; 2 Mechanical Engineering, Faculty of Engineering and Physical Sciences, University of Southampton, Southampton SO17 1BJ, UK; XL.Zhang@soton.ac.uk; 3 Pharmaceutical Microbiology Department, Faculty of Pharmacy, Tanta University, Tanta 31527, Egypt; lamia.youssif@pharm.tanta.edu.eg

**Keywords:** SARS-CoV-2, waterborne pathogens, wastewater surveillance, microbial forensics, next generation monitoring tools, lab-on-a-chip, preparedness, RT-LAMP, PCR

## Abstract

The spectrum of emerging new diseases as well as re-emerging old diseases is broadening as infectious agents evolve, adapt, and spread at enormous speeds in response to changing ecosystems. Severe acute respiratory syndrome coronavirus 2 (SARS-CoV-2) is a recent phenomenon and may take a while to understand its transmission routes from less traveled territories, ranging from fomite exposure routes to wastewater transmission. The critical challenge is how to negotiate with such catastrophic pandemics in high-income countries (HICs ~20% of the global population) and low-and middle-income countries (LMICs ~ 80% of the global population) with a total global population size of approximately eight billion, where practical mass testing and tracing is only a remote possibility, particularly in low-and middle-income countries (LMICs). Keeping in mind the population distribution disparities of high-income countries (HICs) and LMICs and urbanisation trends over recent years, traditional wastewater-based surveillance such as that used to combat polio may help in addressing this challenge. The COVID-19 era differs from any previous pandemics or global health challenges in the sense that there is a great deal of curiosity within the global community to find out everything about this virus, ranging from diagnostics, potential vaccines/therapeutics, and possible routes of transmission. In this regard, the fact that the gut is the common niche for both poliovirus and SARS-CoV-2, and due to the shedding of the virus through faecal material into sewerage systems, the need for long-term wastewater surveillance and developing early warning systems for better preparedness at local and global levels is increasingly apparent. This paper aims to provide an insight into the ongoing COVID-19 crisis, how it can be managed, and what measures are required to deal with a current global international public health concern. Additionally, it shed light on the importance of using wastewater surveillance strategy as an early warning practical tool suitable for massive passive screening, as well as the urgent need for microfluidic technology as a rapid and cost-effective approach tracking SARS-CoV-2 in wastewater.

## 1. Introduction

The emergence of the COVID-19 pandemic, caused by a novel coronavirus (SARS-CoV-2), requires extraordinary measures to deal with this global challenge. There are various factors that have contributed to its far-ranging spread, including enhanced human-to-human transmissibility, sanitation and hygiene, ecological modifications, microbiological adaptation, human susceptibility to infection, human demographics and behaviour, international trade & travel, poverty and social inequality, inadequate public health measures, climate change, and population density (number of individuals/sq km) [1,2,3,4]. Coronaviruses are classified into four genera (alpha, beta, gamma, delta), each one with multiple species with SARS-CoV being divided into different strains [5]. They constantly circulate in humans, and out of the seven known members of the group, four endemic human coronaviruses (HCoV-229E, -NL63, -OC43, and -HKU1) cause mild respiratory, enteric, hepatic, and neurological diseases [6]. In contrast, two relatively recent epidemic strains, severe acute respiratory syndrome coronavirus 1 (SARS-CoV-1) and Middle East respiratory syndrome coronavirus (MERS-CoV) caused severe respiratory disease or pneumonia [7,8,9,10,11]. The SARS-CoV-1 outbreak of 2002–2003 was the first small-scale human pandemic wake-up call of the 21st century, with a mortality rate of approximately 10%, climbing to about 50% in the elderly population; this rate is higher than many other viral diseases [12].

With this background, COVID-19 caused by a seventh member of the coronavirus group (severe acute respiratory syndrome coronavirus 2, SARS-CoV-2) has emerged as a threat to global human health & the economy due to its widespread human-to-human transmission [13] Therefore, understanding SARS-CoV-2′s environmental niches and ecological adaptation for its transmission through the aerial route (coughing & sneezing), exhaled air in the hospital or community settings in association with asymptomatic carriers [14] and exposure through fomites [15,16] to hospital wastewater [17,18], or sewers could be considered as a risk factor in gauging future risk assessment and developing epidemiological models [19]. SARS-CoV-2 can persist in various environments as well as on different surfaces [20]. For example, it is viable on stainless steel or plastic surfaces for 48–72 h until reduction occurs [15]. Moreover, the virus persists for 4–5 days on glass, PV, silicon rubber, and surgical gloves [21,22]. Copper surfaces are known to damage the virus [15]. Additionally, high temperatures (70 °C) interferes with viral persistence while resists low temperatures (22 °C) for 14 days [23,24,25]. The viral persistence seems not to be statistically influenced at different pH values [25]. A higher humidity rate enhances viral persistence [25]. SARS CoV-2 (with variable average mortality rates ranging from <1 to >10%), like SARS-CoV-1 of 2003 (with a mortality rate of approximately 10%) and MERS-CoV of 2012 (with a mortality rate of approximately 35–40%), are transmitted by air or direct contact, but their transmission through water has not been thoroughly explored and investigated [26,27,28,29]. However, transmission through wastewater has been better elucidated for another member of the group, human enteric coronavirus (HCoV-OC43), which is associated with necrotizing enterocolitis and gastroenteritis [9,30,31]. SARS-CoV-2 discharged from faecal (10^2^ up to 10^8^ RNA copies per gram which can be secreted for 14–21 days) [18,32] and urine specimens have been successfully cultured in Vero E6 cells [33,34] and the release of the virulent virus into the gastrointestinal tract indicates the potential faecal-oral transmission path [35], which needs further investigation in order for it to be established beyond doubt [18,32,34,36].

COVID-19 was declared a public health emergency of international concern by WHO based on the International Health Regulation (2005) in April 2020, and the need for coordinated efforts for a better response was stressed [37]. The COVID-19 emergency not only reminds us of the 1918 Spanish-flu pandemic but also, to some extent, another ongoing global crisis, polio, a crippling human disease caused by wastewater dwelling ancient poliovirus, which may have originated as early as 1580 BC [38]. SARS-CoV-2 appears to persist for a long time, like influenza and poliovirus, and hence, multipronged approaches are required to deal with this pandemic in order to prevent it from rapidly transitioning to become endemic globally. Moreover, more widespread epidemics and new waves are expected in the near future, widening the gap between rich and poor [39]. In order to avoid catastrophic outcomes and to deal with the current pandemic and subsequent epidemics, the global community should enhance massive testing capacity in order to make it affordable for all [40,41].

However, considering the ongoing spread of SARS-CoV-2, there is a dual challenge of tracking it not only in terms of its community transmission but also monitoring its global outreach and penetrance [40,42]. It may not be eliminated from the environment or human ecosystem due to its potential future endemicity [43]. In light of this, the challenge may be addressed not only through effective surveillance strategies but also by implementing the outcomes of intelligent data generation approaches for better preparedness and response [44,45,46]. Large-scale and quick population screening with the higher sensitivity and specificity is key to detecting the community transmission of SARS-CoV-2 [47,48,49]. Hence, it is vital to develop cost-effective, portable, fast testing protocols using a small number of reagents and to employ them to tackle the COVID-19 international global health emergency [50,51,52]. Economic surveillance approaches with broader applications will help to assess the risk of community transmission better and consequently help in implementing subsequent measures by testing, tracking, and isolation, not only to reduce the burden on healthcare systems or hospitals but also to make quick decisions to prevent the spread of localised community transmission of SARS-CoV-2.

Similarly, forthcoming third waves, as well as repeated local outbreaks, in the absence of mass-scale testing or limited capacity for clinical testing, can be predicted by wastewater surveillance by improving existing epidemiological models, addressing various variables such as temperature, humidity, matrix composition, and rainfall [18,53,54,55]. Under the current circumstance, this environmental surveillance could be implemented in wastewater treatment plants as a tool designed to help authorities to coordinate the exit strategy to lift their coronavirus lockdowns gradually. In this regard, risk assessment for detecting viruses from infected/contaminated sites by developing and implementing rapid diagnostic methodologies for point-of-care detection for long-term surveillance is a key challenge. 

## 2. Health Inequities, Environment and SARS-CoV-2

SARS-CoV-2 (COVID-19) has emerged as the most important viral disease of zoonotic origin, already directly affecting more than 82 million and causing more than one million deaths in 213 countries as of 2 January 2021 [56]; it has continued to threaten the world population of 7.7 billion since its outbreak in November–December 2019 in Wuhan, China [57]. It is clearly shown that the outcome of SARS-CoV-2 infection is heavily dependent upon age and comorbidities or underlying health conditions [5]. European countries appear to be among the highest continental risk groups given how effectively and efficiently they responded by putting in place necessary measures to protect from infection, such as travel restrictions, isolation or lockdowns, and extensive testing and tracing [58,59,60]. The statistics suggest that the Case Fatality Rate (CFR) due to COVID-19 varies from 21.5% to 0.29% for Guyana and Iceland, respectively [61]. However, it is mainly seen in the range of approximately 1% to >10% in the majority of countries across the globe [26].

In contrast, recent data have shown that approximately 1.3 billion impoverished people are present in 101 countries, predominantly in Asia and Africa, where challenges of undernourishment, deficiency of one or more critical nutrients, sanitation & hygiene, lack of access to clean drinking water, diarrheal diseases as well as poor healthcare systems are already impacting the livelihoods of their populations [62,63,64,65,66,67]. Moreover, these countries are also ranked high not only in terms of poverty, but they are also considered zoonotic disease hotspots. There tend to be increasing population sizes in the most populated countries in terms of their current growth rate as well as their population density per square kilometre. Examples include 218 people/sq km in Nigeria (annual growth rate of 2.7%), 273 people/sq km in Pakistan (annual growth rate of 2.5%), and 414 people/sq km in India [68]. Similarly, urbanisation trends may possibly further complicate tackling the SARS-CoV-2 challenge.

The healthcare systems in LMICs and HICs show significant disparities with an average of ~1% for Venezuela to >17% for USA of their total Gross Domestic Product (GDP) invested [69], making LMICs somewhat fragile in terms of dealing with any global health emergency for a sustained period. This is further evident from the fact that many cases of preventable diseases ranging from diarrhea, measles, tuberculosis (TB), hepatitis, and polio are on the rise in LMICS [70,71,72,73,74]. Polio, in this regard, is still affecting children despite decades-long extended vaccination campaigns driven by millions of polio workers [75,76]. The recent surge in Pakistan has shown that the numbers of cases are picking up again despite an initial projection that the country would be free of polio by 2018 [77]. In addition, despite an ongoing Global Polio Eradication Initiative (GPEI) endgame strategy, the alarmingly difficult circumstances, prevalent in countries like Pakistan and Afghanistan, have certainly harmed the polio drive; one such difficulty is that polio is not recognised as a significant threat mainly because of conspiracy theories, challenges of transport and delivery of vaccines, sanitation, and hygiene as well as social and religious behaviour [78,79,80,81]. Poor wastewater management and disposal have resulted in extensive poliovirus (PV) environmental surveillance initiatives (analysis of sewage), mapping hotspots, not only to detect reservoirs of wild poliovirus (WPV) but also vaccine-derived poliovirus (VDPV), a potential risk for acute flaccid paralysis (AFP) [82,83,84,85,86].

The burden of COVID-19 is enormous, impacting rich and poor countries across the globe by hitting the global public health and economy hard, most notably in LMICs and among them, in particular, the ones which are ranked high in terms of poverty globally. The resulting impact of COVID-19 on public health and livelihoods is enormous, and this requires mitigation at various levels. This includes coordinated global and local emergency responses at multi-sectoral levels and addressing a range of issues such as public awareness, diagnostic kits, hand sanitizers, facemasks, personal protective equipment (PPE), preparing adequate quarantine facilities and measures, ventilators, and sharing of updated information. WHO, in this regard, initially identified high risk and extremely low income or resource-deficient countries, in particular, African and Southeast Asian countries [87].

The basic reproductive number (R0) estimates the average number of persons that received infections from an infected individual within a completely susceptible population [88]. Previous studies found R0 of SARS to be 2.7 [89] and that of H1N1 influenza pandemic to be 2.4 while the basic reproductive number R0 was 2.2 [90]. The re-emergence of SARS-CoV-2 with a high R0 requires integrated early warning and response systems. However, based on the fact that the policies and decision-making are guided by the infection fatality rate (IFR), calculations based on sero-prevalence data may give a clue as to the total infected population [91]. This would certainly help in better coping with the influx of patients and would also lead to a significantly reduced number of healthcare providers and healthcare workers contracting infections and dying [92]. About 17 years ago, we witnessed the loss of $40–50 billion in the global economy and ~$20 billion in Asian countries due to SARS-CoV-1, which was later successfully eliminated by rigorous contact tracing and implementing strict case isolation measures; no further cases have been reported since 2004 [93,94]. In January 2020, WHO first announced that COVID-19 was a world health emergency [95]. Since this date, the emergency has evolved into an economic crisis and global public health concern that has affected the global economies to the tune of $90 trillion, beyond anything experienced in nearly a century [96].

## 3. Transmission, Tracking & Assessing the Burden of Disease

There has been a significant contribution of aerosols in the spread of notorious respiratory viruses such as the influenza virus, SARS-CoV-1, and MERS-CoV [97]. The current SARS-CoV-2 and its infectiousness, along with the infectiousness of other similar diseases or the presence of trained immunity, could vary with conditions such as a change in humidity and temperature [15,98,99,100,101,102,103]. If the infected individual sneezes or coughs, the virus-bearing aerosols discharged will be inhaled by the respiratory tract of another individual, and if the droplets are very fine in size and adequate in number, they are highly transmissible and infective and likely to establish full-blown infection [104,105,106]. Moreover, studies have also reflected that the viral infectiousness is at its peak just before symptoms appear or at its onset due to the higher viral load in the upper respiratory tract [55]. However, recently there have been conflicting views expressed on the transmission levels of SARS-CoV-2 by asymptomatic carriers [53].

The currently developed methods for testing and tracing lack sensitivity and specificity because of minor details from sample collections, and their preparations, variable viral load, faulty primer/probe design, and mutations or polymorphism resulting in polymer chain reaction (PCR) failure [14,107,108,109,110]. Consequently, the reverse transcriptase-quantitative polymerase chain reaction (RT-qPCR) assay of pharyngeal swab samples shows a positive rate of only 28.2% [111]. Moreover, the study reflected that primer sets tend to amplify even in the absence of the template cDNA with a Ct value ~20 [109]. In contrast, false-negative detection has been observed in patients suffering from COVID-19, questioning the reliability and sensitivity of detection [32,112] along with its expensive and labour-intensive. RT-PCR-based assays usually have a better sensitivity [109]. It depends on preparing samples using specific treatment followed by RNA extraction and cDNA preparation, amplification of target genes and detection [32,113,114,115]. However, they have inherent limitations being lengthy, technically demanding, and somewhat non-consistent due to variation in viral load, resulting in false-negative and false-positive outcomes [14,116]. 

The emergence of COVID-19 has exposed our healthcare systems, and associated disparities and disease remind us, to some extent, of the initial days of polio in history where the virus was firmly entrenched in a global environment through wastewater and due to its subsequent spread through the fecal-oral route [117,118]. In addition, it means that polio eradication will be most likely to be affected in particular, which has been a crucial component of the GPEI prior to the COVID-19 emergency in the remaining polio-infected countries, i.e., Pakistan and Afghanistan; this is alongside infections like measles in countries like India, Pakistan and Nigeria with already relatively lower vaccine coverage and poor follow-up records for routine immunisation programmes [119,120,121,122]. SARS-CoV-2, a respiratory virus, has also been shown to persist in the gastrointestinal tract as evident from its presence in the faecal samples of COVID-19 patients, who have tested positive for SARS-CoV-2 RNA [35]. Furthermore, its association with protein receptor or Angiotensin-converting enzyme 2 (ACE-2) [123,124] and release of infectious virions from the virus-infected gut through faecal discharge, in large numbers, is a worrying sign [125,126]. Studies further suggest that viral samples are extensively released into wastewater through faecal shedding and can last for longer even in those patients who have been declared PCR negative in oral & throat swabs and are not showing any COVID-19 respiratory symptoms even after a month [32,127]. There is currently limited information on the environmental persistence of SARS-CoV-2, but since other coronaviruses can remain viable in sewage for up to 14 days, depending on the environmental conditions, it can act as a reservoir, harbouring potentially infectious viral particles [128,129]. 

WHO recommends predicting future disease outbreaks before they reach pandemic levels and early detection among carriers, and the associated environment is one way to do that, in line with the One Health Concept [130,131]. However, lower sensitivity and much more expensive tests ranging from ~$10–40/test), will make it extremely difficult to screen populations, particularly in LMICs. Moreover, a high proportion of patients are diagnosed as false-negatives due to the defective swab sampling that could miss the infected material [132,133]. Considering both the ongoing invasion of SARS-CoV-2 as well as challenges in tracking, SARS-CoV-2 may not be eliminated from the environment or human ecosystem in the near future due to its potential future endemicity. Therefore, the challenge can be addressed through effective surveillance/preparedness. Since quick and large-scale population screening is the key to detect community transmission of SARS-CoV-2 globally, realistic standard testing protocols are vital to assess the risk of community transmission and consequently implement subsequent measures by testing, tracking, and isolation. This will help in reducing the burden on healthcare systems, and making quick decisions to prevent the spread of localised community transmission of SARS-CoV-2 [134,135,136]. Similarly, the forthcoming third waves, as well as repeated local outbreaks in the absence of mass-scale testing or limited capacity for clinical testing, can be predicted by wastewater surveillance by improving existing epidemiological models addressing various variables such as temperature, humidity, matrix composition, rainfall [18,53,54,55].

## 4. Wastewater Screening for Different Viruses

Since the majority of persons infected with gastroenteritis viruses shed large quantities of these viruses in faeces for days or weeks, it is expected that their detection in wastewater could be applied as an early warning of virus outbreaks [137]. Since an infected person excretes between 10^7^ to 10^13^ virus particles daily [138], it would be useful to analyse incoming sewage to detect and quantify excreted human faecal viruses, giving an approximate idea of the number of infected persons.

In the city of Gothenburg, Sweden, a group of scientists performed a study to analyse the presence of seven different enteric viruses (adenovirus, norovirus, Aichi virus, astrovirus, rotavirus, hepatitis A virus, and hepatitis E virus) in wastewater [137]. They found that all viruses except parechovirus could be detected. During the seventh week, they found that there was a peak in the number of detected viral genomes. They found that this week was the winter school holiday week in Gothenburg, indicating that there may have been an influx of individuals from other areas who shed virus during this winter school holiday week. The importance of this study is that it not only detected different viruses in wastewater, but also gave an indication of the movement of infected persons through different areas. In China, during SARS outbreak of 2004, the RNA of SARS-CoV was found in all of the untreated samples 10/10 (100%) and in only 3/10 (30%) of chemically treated wastewater samples obtained from a hospital receiving SARS patients in Beijing [139]. Moreover, molecular detection of SARS-CoV-2 in wastewater was also reported in USA, France, Netherland, and Australia [140,141]. Additionally, a maximum concentration of 10^6^ viral RNA copies per liter was also recorded [142,143,144,145]. 

Wastewater analysis for enterically transmitted viruses has many advantages [137]. For example, researchers can monitor a large population by analysing collected samples in one place [137]. It may also reflect the real magnitude of a virus circulating in the community because wastewater contains enteric viruses excreted from subclinically infected persons as well as ill people [137,146]. Systematic and continuous monitoring of wastewater could give early warning alerts for public health authorities on the ongoing or even future viral outbreak [147]. Furthermore, it is considered as an unbiased method for assessment of infection spread in different areas, particularly where resources essential for clinical diagnosis are limited and can also detect low viral levels [148]. It was also reported that the virus could be shed in faeces before the onset of symptoms [149], which is one to two days for norovirus GII and four to five days for astrovirus [150], while for hepatitis A and E viruses, several studies reported that there could be a more extended excretion period of up to seven weeks [151]. Many virus detection techniques in sewage have evolved for poliovirus detection in line with the WHO polio eradication program [152,153,154]. The study of Ahmed et al. [148] reported the numbers of SARS-CoV-2 RNA copies detected in untreated wastewater and hence estimation of the number of the infected persons. 

In the UK, a group of researchers monitored enteric viruses (adenovirus, JC polyomavirus, noroviruses, sapovirus and hepatitis A and E viruses) from wastewater sources to beaches and shellfish beds for one year [155]. They reported that both adenovirus and JC polyomavirus were found in the majority of samples without seasonal patterns while Hepatitis A and E viruses were not detected. Moreover, Noroviruses and sapovirus were detected at high concentrations and their detection was correlated with local gastroenteritis outbreaks, suggesting that some pathogenic viruses can be directly monitored as a way to prevent future outbreaks.

## 5. Wastewater Monitoring and Surveillance for SARS-CoV-2

Based on the SARS-CoV-2 transmission patterns, WHO issued guidelines on physical distance (2 m) and wearing masks, initially by symptomatic patients and later by all in public places, and by implementing lockdowns [156,157,158]. There has been confusion due to insufficient knowledge on virus transmission, which has undoubtedly led to indecisive and ineffective mitigation strategies and policies triggering the propagation of the COVID-19 pandemic [159,160]. Long-term, inexpensive surveillance of SARS-CoV-2 would help public health agencies to implement appropriate measures and governments to shape their economies, and the key to that is employing cost-effective and non-invasive surveillance strategies [161]. Environmental microbiologists have investigated pathogens such as waterborne, foodborne and faecal-oral viruses or enteric viruses such as norovirus, hepatitis A virus, and poliovirus from the sewage excreted through faeces and used it as a public health surveillance tool [137,162,163,164,165,166,167,168,169], and more recently for monitoring SARS-CoV-2 [115,140,142,144,170,171,172,173,174,175].

SARS-CoV-2 can resist standard disinfection treatments such as sodium hypochlorite and at an eco-friendly reduced concentration of free chlorine [176,177,178]. This is reflected by high levels of SARS-CoV-2 (0.05–1.87 × 10^4^/L present in wastewater even after treatment with sodium hypochlorite, perhaps due to the virus being embedded in faecal particles [179] or in association with other resistant microbes [180]. This may lead to leakage of the virus and its spread through drainage pipelines on a larger scale [179]. Moreover, the hitchhiking of non-enveloped and enveloped viruses along with the coexistence of a relatively resistant plethora of microbes or bacteria (e.g., *Escherichia coli* and f2 phage) may help their better survival by aiding them to tolerate chemicals or disinfectants such as lipid solvents, chloroform and to tolerate a relatively broad range of pH and temperature [34,181,182,183].

It is unprecedented in history that global economies and communities have so extensively relied on the availability of cost-effective rapid, and reliable testing methods, as they are for SARS-CoV-2; such a response allows governments to devise timely intelligent strategies for effective responses. This rigorous contact tracing helped countries like Taiwan, Singapore, and South Korea to avoid lockdowns [184]. Global economies can only recover up to their full potential when all the economic and industrial growth sectors and the masses are convinced that the risk of transmission of SARS-CoV-2 has been marginalised, or at least, we know with precision where it is circulating [185,186,187]. In this regard, the economies which are predominantly relying on selected lockdowns and relaxing them without any concrete data will result in even bigger surges impacting both economies and public health [188].

Environmental or wastewater monitoring is an effective tool for passive mass screening or surveillance generating useful data for early warning against pathogens such as SARS-CoV-2 [161,189,190]. Studies have just begun to investigate the environmental factors controlling the distribution and abundance of SARS-CoV-2 [191,192,193]. However, the importance of this linkage is well understood in the context of poliovirus. SARS-CoV-2 has been reported in many studies to be circulating in medical wastewater [179], septic tanks [179], and wastewater [142], in a situation similar to diarrhoea-causing HCoVA-OC43 [9,179]. Wastewater surveillance can facilitate properly timed and targeted shutdowns and reopening in specific densely populated geographic areas that lack, in particular, means and resources [194,195]. This can be facilitated by using viral concentration methods for wastewater samples in order to increase the sensitivity of the generally used tests [196,197]. These methods include ultracentrifugation, the use of electropositive and electronegative membranes, and polyethylene glycol precipitation [148,196,197,198,199]. 

There is an urgent need to perform cost-effective epidemiological surveillance studies [200,201]. Although massive RT-qPCR testing campaigns are being launched in several countries to monitor the actual prevalence of SARS-CoV-2, this is not a practical surveillance approach for the general population over the long term [200]. Several studies have reported that coronaviruses had been involved in nosocomial outbreaks with environmental contamination as a possible route of transmission; these include a recent study that found nosocomial transmission of SARS-CoV-2 [202]. Nevertheless, the extent of environmental contamination and the mode of transmission are still largely unknown. In a recent study, a patient suffering from upper respiratory tract infection without clinical signs of pneumonia had two positive stool samples for SARS-CoV-2 on RT-PCR despite not having diarrhoea, indicating that viral shedding in the stool could be a possible transmission route [203].

Therefore, it is important to gather information about the presence and future destiny of SARS-CoV-2 in sewage to assess the possible risk to sewage workers and the population at large, and to assess if sewage surveillance is a suitably sensitive tool for monitoring SARS-CoV-2 in the community. In Spain (city of Valencia), a team of scientists and engineers are accessing the sewage network in an attempt to find out where COVID-19 outbreaks are likely to spring up next [204]. Several studies found that sewage surveillance may act as an early alarm for the emergence of COVID-19 in communities, similar to the poliovirus sewage surveillance, which has been used for this goal [141,144]. Most studies, which are performed to assess the presence of SARS-CoV-2 in sewage, are based on detection of the virus by making RT-qPCR or nested RT-PCR, after specific treatment of sewerage samples [113,115,142,200]. Furthermore, Randazzo et al. [200] consistently detected SARS-CoV-2 RNA in samples taken when communicated cases in that region were only incipient. They also found that the wastewater viral RNA context remarkably increased and suggested the subsequent ascent in the number of declared cases. They strongly suggested that SARS-CoV-2 was undergoing community transmission earlier than previously believed, indicating that wastewater analysis is a cost-effective and sensitive tool for COVID-19 epidemiological surveillance.

Medema et al. [144] reported the first detection of SARS-CoV-2 in sewage in the Netherlands. Although they found that COVID-19 prevalence was low, the detection of the SARS-CoV-2 in sewage indicates that sewage surveillance could be a sensitive tool in monitoring the circulation of the virus in the population. In France, Wurtzer et al. [115] proposed that quantification of SARS-CoV-2 genomes in wastewater should be in agreement with the number of non-symptomatic or symptomatic carriers. They also aimed to study the impact of lockdown on the SARS-CoV-2 in wastewaters, so their study was performed from 5 March to 23 April 2020, thus including the period of lockdown in France (from 17 March 2020). They confirmed that the rise in the genome units in raw wastewater perfectly followed the rise in human COVID-19 cases seen at the regional level. SARS-CoV-2 genomes could be detected before the beginning of the exponential growth of the epidemic [115]. They detected a noticeable decrease in the quantities of genomes units simultaneously with the low number of new COVID-19 cases which was an expected outcome of the lockdown. They suggested that quantitative monitoring of SARS-CoV-2 genomes in wastewater should give further and pivotal information for better surveillance of SARS-CoV-2 circulation at the local or regional scale. In Pakistan, Sharif et al. [175] found that 21 wastewater samples (27%) from 13 districts were PCR positive, indicating that wastewater surveillance has an epidemiologic potential which could be considered to be an early warning system for monitoring viral tracking in different districts. 

Haramoto et al., [205] carried out the first environmental surveillance for SARS-CoV-2 RNA in Japan. They detected SARS-CoV-2 RNA (2.4 × 10^3^ copies/L) in secondary-treated wastewater. Samples collected from influent and river water were negative for SARS-CoV-2 RNA. The remarkable information is that SARS-CoV-2 RNA was detected when the reported community cases were high, implying that SARS-CoV-2 wastewater surveillance may be considered as an ideal surrogate for community cases. Therefore, it is important to have information about the presence and future destiny of SARS-CoV-2 in sewage to assess the possible risk to sewage workers and the population at large, and to assess if sewage surveillance is considered to be a sensitive tool for monitoring SARS-CoV-2 in the community.

## 6. Wastewater Microbial Forensics and SARS-CoV-2

COVID-19 is a continuously looming threat to the world economy, public healthcare, public harmony, and stability and, given the circumstances, wastewater surveillance could be implemented at household levels or community levels/wastewater treatment plants for pooled samples as a tool to help authorities to coordinate the exit strategy for lifting their coronavirus lockdowns gradually. In this regard, risk assessment for detecting SARS-CoV-2 from infected individuals/contaminated sites by developing and implementing point-of-care rapid detection is a crucial challenge. 

The sensitivity of existing wastewater detection assays or their analytical sensitivity (RT-qPCR) may be increased [144,206,207,208], and global standards may help to achieve desirable goals. Furthermore, digital RT-qPCR (dRT-qPCR), earlier used for detection of waterborne pathogens and, in particular, quantification of norovirus in wastewater [209,210,211], has been more recently employed for clinical SARS-CoV-2 samples. dRT-qPCR can be employed with certain improvements, not only for detecting SARS-CoV-2 in complex wastewater sources but also in order to quantify the viral load [114].

Based on the current knowledge, it is challenging to determine how long SARS-CoV-2 virus will be with us, and hence protecting the community in general and the residents of hot spots or high-risk areas, in particular, will be a major global health challenge [212]. SARS-CoV-2 carriers with the presymptomatic condition or paucisymptomatic manifestation usually remain undetected in health surveillance systems and hence are the silent reservoir via which the disease is spread [172,212,213,214,215]. Therefore, comprehensive and cost-effective long term surveillance using wastewater-based epidemiology for SARS-CoV-2 will flourish in the near future [216], and prediction models based on estimated viral RNA copy numbers observed in the wastewater will be used to assess the forthcoming disease burden and disease prevalence using simulation studies [140,166,217,218]. The cost involved in a single round of clinical mass screening, depending on the population size of the country, may range from millions to billions of US$, and wastewater-based epidemiology (WBE) is a hugely cost-effective alternative [145,161].

New methodologies are changing the approach to monitoring pathogens in wastewater and will, therefore, affect our ability in the future to assess risk. At present, it is difficult to establish concentrations of specific, viable, and infective SARS-CoV-2 in wastewater in the way that we can for many other pathogens. The increasingly widespread use and continuing development of RT-qPCR and its application in situ for polymerase chain reaction to the single-infective unit level, magnetic separation, and sample enrichment techniques will undoubtedly improve detection and surveillance of SARS-CoV-2. The critical challenge is how to make these new methodologies cost-effective for SARS-CoV-2 monitoring, particularly in developing countries where resources are extremely limited. Reverse Transcription Loop-Mediated Isothermal Amplification (RT-LAMP) PCR is a rapid and single-step cost-effective technique with enhanced sensitivity and specificity, employing four different primers and potentially exploiting different target sequences resulting in amplicon detection from pink to yellow colour transition (pH indicator) [219,220,221,222]. The current detection limit for colourimetric RT-LAMP ranges up to approximately 120 copies and requires improvement [102,223]. 

Water quality is usually tested using standard faecal coliform and streptococcal (enterococcus) assays, which are used by resource managers as indicators of the sanitary quality of water, to determine how well these standard assays reflect true pathogen risk [224,225,226,227]. Similarly, viral water quality indicators may be useful in regulatory and surveillance applications where faecal material associated bacteriophages or even human viral pathogens such as adenovirus, polyomavirus, norovirus, and reovirus has been used as a water quality indicator [228,229,230,231,232,233,234,235] With the current pandemic, SARS-CoV-2 has certainly become the virus to be investigated across the six continents as a wastewater monitoring tool. However, being an RNA virus, the persistence of SARS-CoV-2 can vary greatly geographically and temporally across the world [161]. The human gut virome associated with health and disease provides an opportunity to exploit viral metagenomics to detect it in excreted faecal material [229], including for SARS-CoV-2, and is combined with the determination of the viral load [161] as previously reported [236,237,238]. Additionally, there are multiple factors that correlate the viral survival in wastewater, including; concentration of suspended solids, temperature, organic matter, and pH [239].

## 7. A Journey from Gut Microbiome to Urban Sewage Metagenomics

It is a well-known fact that the composition of the human gut microbiome or virome alters due to various factors [240,241] as well as with the passage of the disease [242]. Zuo et al. [243] reported that faecal samples with SARS-Co-V-2 viral activity was associated with abundant amount of *Streptococcus infantis*, *Morganella morganii*, *Collinsella tanakaei,* and *C. aerofaciens*. Additionally, the capacity for nucleotide, carbohydrate, and protein metabolism was enhanced. An excreted gut-associated pathogenic virus such as norovirus is found in varying abundance in sewage across the world [244,245,246]. Similarly, another virus called human bocavirus (HBoV), a member of the parvovirus family, persists as potentially both an enteric and respiratory pathogen and can be detected variably in the faecal material of children with stomach flu and has been detected in wastewater or sewage-contaminated drinking water using approaches such as qPCR and Luminex assays [234,247]. Indeed, there is a bidirectional relationship between SARS-CoV-2 and gut microbiome, and both of them influence each other. Gut dysbiosis may result in SARS-CoV-2 translocation from pulmonary to intestinal lumen through lymph as well as circulatory systems [248]. On the other hand, COVID-19 could alter the gut microbiome via the interaction of the “gut-lung axis”, which may interfere in the functioning of these critical organs and may well be linked to the varied susceptibility of different age groups to SARS-CoV-2 [249]. It was also reported that Coronavirus particle integrity can be affected by some bacterial surface molecules such as surfactin, which targets influenza A virus [248]. Severe dysbiosis detected in COVID-19 patients and metabolites produced by gut microbiota affect the immune response leading to inflammation in the lung and disease development [243]. It was reported that pathogens such as *Klebsiella oxytoca*, *Faecalibacterium prausnitzii*, *Rothia mucilaginosa,* and Tobacco mosaic virus were predominant and abundant in COVID-19 subjects [248,250]. Consequently, a dangerous inflammatory environment will influence the lungs through circulation [171].

The limited studies on SARS-CoV-2 have shown the disease severity index changing with the alteration of gut microbiota [243,251,252,253,254]. Moreover, SARS CoV-2 RNA has not only been detected in the faecal material of symptomatic patients but is also seen in association with asymptomatic individuals [55,179,252,255,256]. There is no concrete evidence about the survival of SARS CoV-2 in sewage [257], and its persistence in wastewater may well vary depending on the temperature [161] and hence, its distribution and abundance across six continents may also vary provided other factors are constant. Based on the fact that other members of the coronavirus family can remain viable for 14 days in sewage, SARS-CoV-2 may well persist for that long [128,129].

Therefore, employing metagenomics sequencing (mNGS) and RT-qPCR on pooled wastewater/sewerage samples to assess the community viral load and linking it with the number of people shedding virus may help in assessing risk [161,258,259]. Additionally, genome sequencing provides detailed data regarding the presence of a specific haplotype in certain areas [260,261] stated that raw and treated wastewater samples collected from an Italian plant in Milano were tested for SARS-CoV-2. They mentioned that raw water was positive on the first day while it showed declined positivity after eight days. On the other hand, treated wastewater samples were always negative. Moreover, the viral pathogenicity was non-significant, indicating that wastewater disinfection was efficient or even refer to the natural decrease of viral vitality. These findings were also consistent with the study of Wurtzer et al. [115], who reported that chlorine disinfection before wastewater release was effective in reducing the viral load by 100-fold reduction. This is because the viral concentration might be up to 50–1500 copies/mL in the wastewater inflow [142]. The cost-effective and long-term environment surveillance approach will undoubtedly help. It will reduce the burden on healthcare systems under prevailing circumstances through the setting up of mobile environmental surveillance laboratories for random screening of wastewater or sewerage in addition to identifying hot spots due to symptomatic clusters of patients. SARS-CoV-2 shedding in stool, and its subsequent quantification in wastewater, indeed offers an economic risk assessment and disease management approach within the community through long-term surveillance of wastewater (WBE) [140].

## 8. Next-Generation Monitoring Tools

The frequently increasing episodes of epidemics and pandemics, ranging from SARS-CoV-1, H1N1, MERS, Ebola, and now SARS-CoV-2, have certainly enhanced the desire and impetus of global public health agencies to strengthen their capabilities, including in the area of effective disease surveillance systems for tracking and tracing [87,262,263]. In particular, for LMICs, with relatively low capacity in terms of infrastructure and trained human resources, managing the COVID-19 health emergency is no less than a nightmare.

Nano-biotechnology-based next-generation diagnostics tools or biosensors allow the direct, rapid, and sensitive detection of infectious agents including viruses. Surface-enhanced Raman scattering (SERS) spectra of viruses can be exploited to rapidly differentiate between viruses and virus strains based on a reference library of vibrational Raman fingerprints using tiny volumes (0.5–1.0 µL) [264]. Novel sensor concepts based on nanotubes, nanowires, cantilevers or atomic force microscopy have been applied to diagnostic devices/sensors. These sensors aim to improve the sensitivity, reduce production costs or measure novel analytes, e.g., Bioforce’s Virichip (Ames, IA, USA) uses atomic force microscopy for the detection of whole viruses for early diagnosis of viral infections [265].

Molecular approaches depend on nucleic acid as the main target, and usually do not discriminate between a live pathogen or virus from dead. If DNA is being used for detection, it can persist longer compared to RNA or intact viable virus and detecting “legacy DNA” or “legacy RNA” (from dead or non-infective viruses) may lead to erroneous estimates about the disease burden during passive surveillance. Therefore, such freely floating DNA or RNA can interfere with determining the load of infective material present in wastewater so treating them with DNAase or RNAase or for selective removal of freely floating DNA to enrich RNA can help. To selectively filter out free DNA, intercalating dyes such as propidium monoazide (PMA), activated by light, may facilitate and hence such DNA do not interfere in RNA enrichment and are also not amplified during PCR so only intact virus is amplified [266,267,268,269,270,271]. In addition, the binding affinity of specific viral targets can be exploited for environmental applications in general (enteric viruses—rotavirus and norovirus captured using porcine mucin) and for SARS-CoV-2 in particular. Miniature devices such as “Microfluidic devices” to enrich SARS-CoV-2 using minimal emerging cultivation approaches, direct RNA isolation and associated LAMP reaction for real-time surveillance of viable virus or viral load in wastewater can be employed [271,272,273,274].

Therefore, a rapid, highly sensitive, and accurate diagnostic technique is required to detect the presence of SARS-CoV-2 not only in patients’ samples but also in wastewater, which involves the combination of diverse interdisciplinary fields to develop an armamentarium of tools for WBE [275,276]. A multipronged approach involving a broad range of disciplines: biotechnology, chemistry (analytical chemistry, immunochemistry, biochemistry), microbiology, wastewater treatment operators, infectious diseases, epidemiology, virology, bioinformatics, genomics & evolution, environmental and civil engineering, materials sciences, computer sciences, electrical engineering, nanotechnology and risk communication to develop novel diagnostic systems, is required.

The presence of SARS-CoV-2-like enteric viruses in wastewater is a potential health risk and also an opportunity to develop innovative and economic approaches for wastewater surveillance to address broader public healthcare interests. A complex wastewater sample with variable matrix composition can be processed using customised nano-filtration membranes [277] to enrich SARS-CoV-2, which is a large, positive, non-segmented, enveloped single-strand RNA virus with a spherical, elliptical or pleomorphic form with an average diameter of 60–140 mm [278]. MS2 bacteriophage has been widely used as a surrogate for pathogenic waterborne viruses (poliovirus, hepatitis A virus and perhaps for rotavirus) [279,280,281] to test various nano-filters; it can be modified for SARS-CoV-2 retention and binding to the channels in a “Microfluidic chip”. For instance, carbon nanotube filters were used to eliminate waterborne viral and bacterial pathogens. Therefore, nanotechnology can be used to produce nano-filtration membranes removing viruses and water-borne pathogens which are generally larger than 50–60 nm. The encapsulation of nanoparticles in membranes allows for the combination of several removal processes, e.g., via filtration or selective enrichment. The fabrication of the membrane surfaces, with nanoparticles/nanotube/nanocomposite or biological nano-filters or combined use of membranes and nanoparticles [282], can either selectively interact with the virus or control the pore size of multiple layers to provide a platform for filtration and virus enrichment. 

Several studies have suggested that sewage surveillance for SARS-CoV-2, like poliovirus, may help in risk assessment for the surge of COVID-19 in communities [141,144]. The currently used approaches to detect SARS-CoV-2 in sewage are based on detection of the virus using RT-qPCR [32,113,114,115]. In short, isolation of targeted genome or nucleic acid requires various time-consuming, labour-intensive, and technically demanding high skills that are lacking in resource-deficient settings. Therefore, there is undoubtedly a great deal of work required to develop new integrated approaches [229] and standard protocols for long-term bio-surveillance of the reservoirs of SARS-CoV-2 in wastewater and microbial forensics, which uses the principles of science & technology and other technologies such as microfluidic devices to generate real-time qualitative and quantitative data about SARS-CoV-2. Alongside geographical information, this will be the future diagnostics armaments researchers should be looking forward to developing. Microfluidic integrated cassettes (“chips”) for processing samples and analysis to facilitate point-of-care (POC) immunoassays and nucleic acid-based amplification tests have been shown as innovative approaches [283,284].

A previous study developed a microfluidic cassette performing three critical steps, i.e., separating viral particles-filtration or flow-through, extraction of RNA, making of cDNA and exponential enzyme-mediated isothermal amplification using Nucleic Acid Sequence-Based Amplification (NASBA) and finally the amplified products visualised through DNA probes conjugated with horseradish peroxide for colourimetry [285]. The smart device combining all features with the LAMP-based amplification and real-time optical detection was reported by Mauk et al. [284]. The microfluidic device, with the integrated RNA isolation (influenza virus clinical specimens) approach via microfluidic oil-water interfaces, has recently been employed as a point of use (POU) tool for diagnostics [286]. There are many versions of such microfluidic cassettes or chips (acrylic and polycarbonate) for nucleic acid-based tests or immunoassays with suitable fabrication techniques allowing the storage of reagents, fluid actuation, and flowing control [283]. Integrating the isothermal LAMP amplification reaction with the microfluidic chip using small volumes, i.e., 1 μL reaction volume for each assay, and measuring fluorescence intensity using a smartphone is not only an economical but also a time-saving option [274] compared to available commercial kits for SARS-CoV-2 to assess viral load in sewage samples. A recent study has demonstrated the capability of the microfluidic chip in terms of detecting not only different viral etiologies (dengue, Zika, and chikungunya) but also viral load as well, in clinical samples, and further hooking it up with the mobile information relaying technology for better healthcare management [287]. Further automation in processing SARS-CoV-2 samples, using RT-LAMP isothermal assay, performing equally better as that of RT-PCR, was introduced by integrating swabs with virus spiked synthetic nasal fluids in a viral transport medium (VTM) and subsequent amplification without RNA extraction using a small volume in an RT-LAMP with greater sensitivity, was performed (Limit-of-Detection-50 RNA copies/µL) and in a short time [287].

Glyconanodiagnostics, relying on the unique glycoprotein signatures of SARS-CoV-2, e.g., spike protein (S1 and S2- S1 subunit containing a receptor-binding domain-RBD) and envelope protein, can be potentially exploited in SARS-CoV-2 diagnostics using microfluidic chips as well [288,289]. Glycosylation of a viral protein is mostly higher than cell protein glycosylation which serves as a clear difference between host and viral proteins, and the glycoproteins displayed on the surface of SARS-CoV-2 [290,291] can act as a unique signature tag for its identification and can be exploited using specific binding molecules such as lectins-attached with a colourimetric reagent or fluorescent molecule.

## 9. Human Ecosystem, Preparedness & Disease Management

According to the recent estimate, about 4/5th of the global population (~8 billion) live in developing economies, and 54% of that population resides in an urban area and this figure will likely exceed 66% by 2030 [292]. The urbanisation trends in LMICs, in particular Africa and South Asia, where the healthcare systems are fragile, are faster and the concomitant [293] associated with the increasing population density per square kilometre provides a perfect environment for the broad spread of infectious diseases like SARS-CoV-2. Therefore, the urban environment, which has already become the most complex human habitat, along with the current SARS-CoV-2 pandemic, means that COVID-19 disease management will require extraordinary measures from an environmental and public health perspective as an international health emergency on a global scale. 

It is envisioned that the recent paradigm shift in our understanding will ensue with the routine testing of wastewater treatment facilities, for the emerging viral disease COVID-19, as an early warning system and will help public health departments in informed decision-making. The credibility of the surveillance can be gauged by the improved sensitivity of the tests where a single introduction of infection could be detected in a wastewater reservoir from a community [128,147]. Therefore, testing wastewater regularly, not only for the presence or absence of SARS-CoV-2, but also to determine the viral load or amount of corresponding genetic material will be able to give insight about hotspots for days or weeks for COVID-19. The wastewater surveillance data generated will certainly give public health teams and hospital management breathing space for better preparedness and educated responses, and government can reinforce strict social distancing measures by imposing smart lockdowns. Moreover, the pooled information retrieved regarding the health status of the community, in particular for viruses such as polio and SARS-CoV-2, can thereafter reinforce prioritisation of vaccination and, if necessary, testing, tracing, and isolation in identified hotspot zones vis a vis the ongoing SARS-CoV-2 pandemic. As a matter of fact, one of the better ways to undertake surveillance, in particular in those countries where testing capacity has not been matching the population size, is to simulate or predict the transmission patterns, and infection rate through regular wastewater surveillance [161]. For example, the wastewater-based monitoring system for polio has been in place since 1989 as an early warning system against pathogen reintroduction, and it helped, for instance, in 2013, in better preparedness and launching a subsequent educational response at hotspots in Israel through vaccination of a vulnerable population [258,294,295]. There are only limited numbers of countries or coalitions which have reported SARS-CoV-2 RNA in wastewater and developed a well-planned WBE programme for COVID-19, mainly Australia, Canada, and Europe [144,296,297,298] with limited implementation in the USA [299].

In addition to the increasing interest of the global scientific community in the fields of epidemiology, diagnostics, and clinical medicine for COVID-19 in the last six months or so, the interest in WBE as a non-invasive early warning predictor or monitoring tool for infectious diseases, including SARS-CoV-2, has already tremendously increased and will further increase in the near future [115,140,141,142,144,169,261,300,301,302,303,304]. It will help to minimise the risk of relaxing restrictions or lockdowns too soon or help in imposing intelligent small lockdowns. This is supported by the recent Italian study where WBE was successfully employed to investigate the spatial and temporal patterns of viral spread among the population [169]. 

In order to avoid a catastrophic outcome and to deal with the current pandemic and subsequent epidemics, the global community not only should enhance massive testing capacity so that it is affordable for all (~1 USD/test) but should also be thinking of scaling up the vaccine supply to meet the demands of billions of doses (~8 billion) as soon as it is available. Vaccines are considered to be the ultimate panacea against SARS-CoV-2 where some vaccines have already developed and others still at various stages of development. On the contrary, relying entirely on herd immunity may well be very costly as, in order for this to be effective in any country, the majority of the population, 70% to 90%, has to be naturally exposed to SARS-CoV-2 and recover or build immunity or it must be achieved through vaccination which became available for some types of vaccines. Therefore, in the process of building heard immunity on such a large scale, there will be fatal consequences for a vulnerable population (old age or with underlying conditions) within the global community as has been seen in countries like Sweden and in the crippling of healthcare systems. The operational scale of such a huge supply chain can be gauged from the fact that in 2018, approximately 116.3 million infants were immunised globally for polio (DPT) [305], whereas one billion doses are produced and used annually. It is highly likely that widening gaps of significant proportions for the population not vaccinated for SARS-CoV-2 will create a huge disparity with the result that COVID-19 epidemics will hit many countries around the globe hard and impact the central dogma of One health, hence influencing the global economy (One-Health ⇔ One-Economy). This has been shown, for instance, by economic modelling, visualising a polio-free world with the gain of at least USD 40–50 billion predominantly in LMICs in addition to mitigating the deadly consequences of terrible lifelong disease [306]. 

It is believed that monitoring of water resources, including wastewater, reveals the presence of a broad array of pathogenic microorganisms including PV, SARS-CoV-1 and SARS-CoV-2 [128,143,183], which may be introduced by faecal shedding, freshwater runoff from sewers, rivers, and streams [301,307]. Therefore, PV and SARS-CoV-2 are certainly linked not only with personal sanitation & hygiene but also with the overall human health ecosystem as well as the environmental ecosystem. In addition, both PV and SARS-CoV-2 are single-stranded, positive-sense RNA genomes, and both can exist either symptomatically, with a similar incubation period, and also circulate asymptomatically or silently within-population [128,129], heavily relying on human behaviours. In order to better develop epidemiological models of disease spread in diverse environments, such as those prevailing in HICs and LMICs, it will be essential to trace the circulation of SARS-CoV-2 mostly in wastewater, in particular in hotspots, along the lines of polio environmental surveillance campaigns that have been carried out in the past [258,259].

The COVID-19 pandemic has clearly shown that the global population is highly vulnerable to SARS-CoV-2, which is amplified by environmental drivers. Its transition from pandemic to epidemic to endemic can only be gauged, managed, and understood by probing the environment, especially wastewater. The examination of high-risk groups and composite human faecal samples using environmental surveillance has helped in determining high-risk groups or pockets for polio, and hence such supplementary surveillance is mandatory for maintaining polio-free status [259]. A similar approach should be adopted for SARS-CoV-2 along with enhanced efforts not only to improve campaign quality, penetrating to remote and difficult-to-access areas, but also to screen in the most populous areas. Wastewater surveillance or WBE has become more relevant in particular for LIMCs and highly dense populations in urban settings where testing and tracing for SARS-CoV-2 is either economically not feasible [161] or not possible because of lack of depth in their diagnostic (testing-tracing) capacity. For such low-income settings, poverty-stricken regions or hotspots, WBE can help to assess the disease burden and support timely implementation of mitigation strategies and help LMICs, in particular, to avoid the worst of economic recession by easing lockdowns based on real data and restoring livelihoods for marginalised global communities.

The emergence of COVID-19 has exposed not only global healthcare systems but also their preparedness to deal with the challenges of infectious diseases. Indeed, understanding the epidemiology of disease & corresponding population behaviour in a highly polarised world, i.e., high-income countries (HICs) & low & middle-income countries (LMICs), will help us to understand the transmission conundrum of such once a millennium virus in future. The interdisciplinary approaches will certainly be at the forefront to help nations better prepare for assessing the current pandemic risk or dealing with the third wave or surges of COVID-19 as we move further. Multipronged approaches will be required, ranging from preparedness and national action plans to developing and implementing technologies for surveillance. A microfluidic chip technique integrated with geographic information for real-time surveillance of SARS-CoV-2 in wastewater channels to assess the risk by developing epidemiological models will be of significant value. Large-scale and quick population screening with greater sensitivity and specificity is key to detecting community transmission of SARS-CoV-2. Hence, it is vital to develop cost-effective portable, fast testing protocols using a small number of reagents and for these to be employed for the COVID-19 international global health emergency. Microfluidic cassettes manipulating small amounts of fluids using the channel at the micron-level not only offer broader applications in diagnostics and environmental surveillance but are also cost-effective, portable, rapid, and simple to use. The innovative, refined approaches can further speed up the development and availability of SARS-CoV-2 wastewater diagnostics by modifying conventional clinical laboratory benchtop tests, and hence, help to deal with the COVID-19 emergency with better information content.

## 10. Conclusions

SARS-CoV-2 represents an ongoing challenge to all countries, including HICs and especially LMICs. Its catastrophic impact on economies and health requires us to instantly increase our awareness for better preparedness at the local and global levels. Massive RT-qPCR testing campaigns are being started in different countries to monitor the actual prevalence of SARS-CoV-2. Nevertheless, this is not a practical surveillance strategy for the general population in the long term because of many limitations, such as the fact that it is laborious, lengthy, and somewhat inconsistent due to variation in viral load, resulting in false-negative and false-positive results. Traditional wastewater-based surveillance for poliovirus may offer a lesson on how to deal with SARS-CoV-2. Current studies have reported that wastewater monitoring is considered to be an effective tool for passive mass screening or surveillance, generating useful data for early warning against SARS-CoV-2. Therefore, there is an urgent need for long-term wastewater surveillance as well as developing early warning systems for SARS-CoV-2.

Isolation of nucleic acid using traditional methods is laborious and time-consuming because it requires many washing steps, centrifugation, and different reagents that are not readily available in most of the clinical labs where accurate and rapid diagnostic tools are urgently required. Microfluidic devices have recently been used as point-of-care devices to isolate nucleic acid rapidly. Microfluidic technology can help in developing rapid and cost-effective approaches for the detection of SARS-CoV-2. Novel detection using this microfluidic technology will pave the way for detecting and investigating community transmission of COVID-19.

Tracking SARS-COV-2 in wastewater could give public-health officials a head-start in determining whether to impose restrictions such as lockdowns, wearing masks, and social distancing, etc. Therefore, earlier identification of the virus’ arrival in a community may help in reducing the economic and health burden caused by COVID-19. 

## Data Availability

Data sharing is not applicable to this article as no datasets were generated or analysed during the current study.
